# Foliar functional and genetic variation in a keystone Hawaiian tree species estimated through spectroscopy

**DOI:** 10.1007/s00442-023-05374-1

**Published:** 2023-05-12

**Authors:** M. M. Seeley, E. A. Stacy, R. E. Martin, G. P. Asner

**Affiliations:** 1 Center for Global Discovery and Conservation Science, Arizona State University, Hilo, HI 96720 USA; 2 School of Geographical Sciences and Urban Planning, Arizona State University, Tempe, AZ 85281 USA; 3 School of Life Sciences, University of Nevada, Las Vegas, NV 89154 USA

**Keywords:** Spectroscopy, Plant evolution, Leaf spectra, Genetic diversity, Hawaii, *Metrosideros polymorpha*

## Abstract

Imaging spectroscopy has the potential to map closely related plant taxa at landscape scales. Although spectral investigations at the leaf and canopy levels have revealed relationships between phylogeny and reflectance, understanding how spectra differ across, and are inherited from, genotypes of a single species has received less attention. We used a common-garden population of four varieties of the keystone canopy tree, *Metrosideros polymorpha*, from Hawaii Island and four F1-hybrid genotypes derived from controlled crosses to determine if reflectance spectra discriminate sympatric, conspecific varieties of this species and their hybrids. With a single exception, pairwise comparisons of leaf reflectance patterns successfully distinguished varieties of *M. polymorpha* on Hawaii Island as well as populations of the same variety from different islands. Further, spectral variability within a single variety from Hawaii Island and the older island of Oahu was greater than that observed among the four varieties on Hawaii Island. F1 hybrids most frequently displayed leaf spectral patterns intermediate to those of their parent taxa. Spectral reflectance patterns distinguished each of two of the hybrid genotypes from one of their parent varieties, indicating that classifying hybrids may be possible, particularly if sample sizes are increased. This work quantifies a baseline in spectral variability for an endemic Hawaiian tree species and advances the use of imaging spectroscopy in biodiversity studies at the genetic level.

**Supplementary Information:**

The online version contains supplementary material available at 10.1007/s00442-023-05374-1.

## Introduction

Genetic diversity of forests provides a foundation for resilience to climate change, biological invasions, and other anthropogenic threats (Crutsinger et al. 2008; Schaberg et al. 2008). High genetic diversity of overstory forest species has been linked to increased productivity and fitness (Aravanopoulos and Zsuffa 1998; Arcade et al. 1996; Jelinski 1993; Knowles and Grant 1981; Mitton et al. 1981), a higher tolerance to pollutants (Bergmann and Hosius 1996; Müller-Starck 1985; Oleksyn et al. 1994), and cascading trophic effects on arthropod (Johnson et al. 2006) and fungal (Tang et al. 2022) biodiversity. As genetic diversity is a basis for adaptation and enhanced resilience, it is vital to preserving forest ecosystems, yet anthropogenic disturbances have resulted in significant declines in forest genetic diversity, reducing the future resistance of affected species (Schaberg et al. 2008). While the 15th Sustainable Development Goal of the United Nation includes aims to stop biodiversity loss, including loss of genetic diversity (Le Blanc 2015), the resources with which to quantify and map genetic diversity are constrained because genetic analyses of forest species require extensive field and lab work (Walters and Scholes 2017).

Remote sensing, in particular imaging spectroscopy, has emerged as a powerful tool for quantifying biodiversity at large spatial scales to understand drivers of biodiversity and inform protection priorities (Asner et al. 2017; Féret and Asner 2011). Imaging spectroscopy generates high-spectral-resolution data spanning the visible to shortwave-infrared (SWIR; 400–2500 nm) electromagnetic spectrum. Applied to vegetation, spectroscopy captures the molecular constituents of leaves, mediated by leaf structure. Leaf traits such as leaf mass per area (LMA), chlorophyll content, and secondary compounds, among others are an expression of adaptation of a species to its environment (Ordoñez et al. 2009; Wright et al. 2004). While such quantitative traits often have a substantial allelic basis (Hallgren et al. 2003; Marron and Ceulemans 2006) that is predominantly polygenic (Bourgaud et al. 2001; Orians et al. 2000), heritability of leaf traits derived from spectroscopy has not been widely tested. Due to the capability of spectroscopy to capture these traits, spectral variation tracks genetic variation among and within forest stands (Blonder et al. 2020; Cavender-Bares et al. 2016; Deacon et al. 2017; Madritch et al. 2014; Martin et al. 2007). According to the spectral variability hypothesis, the variability of canopy reflectance spectra within an area is positively related to plant diversity (Palmer et al. 2000, 2002). Consistent with this hypothesis, leaf-level spectroscopy has revealed heritable spectral differences within and among species of *Quercus* (oak; Cavender-Bares et al. 2016) and *Dryas* (an Arctic shrub; Stasinski et al. 2021) as well as within populations of *Populus tremuloides* (aspen; Deacon et al. 2017) and *Metrosideros polymorpha* (ohia, Martin et al. 2007)*.* Ploidy levels and genetic varieties of *P. tremuloides* have been successfully classified using canopy-level imaging spectroscopy (Blonder et al. 2020; Madritch et al. 2014). Further, some studies have revealed patterns of leaf spectra consistent with phylogeographic variation within species (e.g., *Quercus oleoides*, *Fagus sylvatica—*European beach*—*and *P, tremuloides*; Cavender-Bares et al. 2016; Blonder et al. 2020; Czyż et al. 2020; Madritch et al. 2014) or among species (e.g., Neotropical trees; McManus et al. 2016). To develop imaging spectroscopy as a tool for characterizing genetic variation at the landscape level, we must first understand how spectra vary within continuous forest stands, including variation among conspecific varieties and their hybrids, especially at fine spatial and taxonomic scales. This gap in our understanding of how spectroscopy captures functional variation challenges conservation agendas that seek to include genetic diversity.

*Metrosideros polymorpha* Gaudich. (Myrtaceae) is an ideal model species for testing the capacity of spectroscopy to characterize functional genetic variation of forest canopies at fine spatial and taxonomic scales. This dominant tree species comprises a large number of vegetatively distinct varieties and races distributed nonrandomly within continuous forests that span environmental gradients and ecotones within the climatically variable Hawaiian Islands (Dawson and Stemmermann 1990; Stacy et al. 2020; Stacy and Sakishima 2019; Treseder and Vitousek 2001). The many forms of *M. polymorpha*, along with the four other species of Hawaiian *Metrosideros*, appear to derive from a single colonization of Hawaii by the genus ~ 2.6–3.9 million years ago (Choi et al. 2021; Dupuis et al. 2019; Percy et al. 2008; Wright et al. 2000). Diversification within this group is largely the result of adaptive radiation associated with Hawaii’s diverse abiotic conditions (Ekar et al. 2019; Izuno et al. 2022; Morrison and Stacy 2014; Stacy et al. 2014, 2020; Stacy and Sakishima 2019). On Hawaii Island*,* the youngest and largest island in the chain, *Metrosideros* occurs continuously (barring deforestation) from sea level to 2470 m above sea level wherever mean precipitation exceeds 50 cm annually (Stemmermann and Ihsle 1993). The *Metrosideros* community on Hawaii Island comprises just four varieties of *M. polymorpha* associated with different environments: *M. polymorpha* var. *incana* (new lava flows at low-to-middle elevations and dry areas)*, M. polymorpha* var. *glaberrima* (mature substrates at all but lowest and highest elevations), *M. polymorpha* var. *polymorpha* (all substrates at high elevations), and *M. polymorpha* var. *newellii* (riparian zones; Dawson and Stemmermann 1990)*.* All taxon pairs can be crossed to make F1 hybrids (Corn 1979; Rhoades 2012; Stacy et al. 2017), and hybridization between varieties occurs to varying degrees where ranges overlap (Corn and Hiesey 1973; Stacy et al. 2016). Thus, *M. polymorpha* on Hawaii Island presents the opportunity to examine the utility of spectroscopy to discern very closely related, co-occurring tree taxa and their hybrids and to examine the expression and differentiability of leaf traits in the hybrids.

Here, we use a common-garden population of the four varieties of *M. polymorpha* on Hawaii Island and their F1 hybrids derived from controlled crosses to address the following questions: Do leaf-level reflectance spectra differentiate the four varieties of *M. polymorpha* on Hawaii Island? Are patterns of spectral inheritance in F1 hybrids distinct and intermediate to those of their parental varieties, as expected for highly polygenic traits? Finally, for a single variety occurring on multiple islands, we ask: do the reflectance spectra differ between common-garden trees from Hawaii Island and Oahu? We include a discussion of the spectral data in light of evidence of differential adaptation of the four varieties to contrasting environments and to islands of different ages.

## Methods

### Common garden population

The 54 reproductively mature trees used in this study were raised from seed at Panaewa Farm, College of Agriculture, Forestry, and Natural Resources Management, University of Hawaii Hilo, located 75 m above sea level on east Hawaii Island. Seeds were derived from controlled crosses in natural populations on Hawaii Island and Oahu (Rhoades 2012; Stacy et al. 2017, unpub. data*)*, supplemented by open-pollinated seeds, and all trees were maintained at the farm for use in studies of life history traits and hybrid fertility (Stacy et al., unpub. data). The 8-to-14-year-old trees represented the four varieties of *M. polymorpha* on Hawaii Island (hereafter designated glaberrima, incana, newellii, and polymorpha; Fig. SI 1), four inter-varietal F1 hybrid genotypes from Hawaii Island, and a single variety (incana) from Oahu (Table 1). With two exceptions, all genotypes comprised trees derived from > 1 site, or trees for which parents were derived from > 1 site in the case of F1 hybrids; the exceptions were individuals of incana from Hawaii Island and Oahu that were derived from controlled field crosses at a single site on each island. All trees were maintained within a 72’ × 35’ coldframe until 2020 when some Hawaii Island-derived trees were outplanted in a common garden adjacent to the coldframe. We assessed the effect of this outplanting on leaf spectra by comparing greenhouse and common-garden trees of incana-polymorpha F1 hybrids following the methods below and found no significant differences. Thus, we determined that outplanting had negligible effects on the spectra, and all samples were combined for analysis of spectra among genotypes.

**Table 1:** *M. polymorpha* varieties and F1 hybrids used in this study

Island	Variety/F_1_ hybrid	Hawaii island varieties	GI Hybrid	IP Hybrid	NP Hybrid	GP Hybrid	Inter-island
Hawaii	Glaberrima	X	X			X	
	Incana	X	X	X			X
	Newellii	X			X		
	Polymorpha	X		X	X	X	
	Glaberrima-incana		X				
	Incana-polymorpha			X			
	Newellii-polymorpha				X		
	Glaberrima-polymorpha					X	
Oahu	Incana						X

Varieties are not highlighted, while hybrids are highlighted. Island of origin is noted, though all individuals were grown in a greenhouse/common garden on Hawaii Island. Groupings used to assess separability with principal component analysis and analysis of variance are noted in columns three through eight with “X” denoting membership in each grouping. Six plants per variety/F_1_ hybrid were included in each grouping

### Leaf measurements

We measured leaf reflectance spectra on six trees from each of the nine genotypes (treating incana from Hawaii Island and Oahu as separate genotypes). A minimum of 11 leaves were collected from each plant, placed in zip lock bags, and stored on ice for transport to the laboratory for analysis within four hours. We selected leaves from sunlit portions of the plant with minimal discoloration (e.g. chlorosis) and sooty mold. Five representative leaves per tree were selected and wiped clean with water and patted dry prior to spectral measurements. Spectral measurements were collected using a leaf clip and field spectrometer at 1-nm intervals from 350 to 2500 nm (Analytical Spectra Devices Inc., Boulder, CO, USA). Spectra were calibrated using a white reference and corrected using parabolic correction to optimize spectrometer measurements (Hueni and Bialek 2017). Parabolic correction was performed to correct for differences in temperature sensitivity of sensors within the field spectrometer. A jump in the spectra often occurs around 1000 nm due to the silicon-based sensors for the visible to near infrared and can be corrected post hoc according to Hueni and Bialek (2017). Finally, the brightness normalization was applied to all spectral measurements, as it minimizes noise (Kruse et al. 1993; Myneni et al. 1989). Reflectance values below 400 nm were removed, as wavelengths between 350 and 400 nm have a low signal-to-noise ratio. Leaf spectra were averaged by plant. Following spectral measurements of all leaves, leaf area was calculated using ImageJ from a leaf scan collected with an EPSON scanner at 600 dots per square inch. Once dried for 72 h at 65 degrees Celsius, leaves were weighed, and leaf mass per area (LMA) was quantified for each plant.

### Analysis

To assess whether leaf spectra can differentiate the varieties of *M. polymorpha* and their hybrids, we used principal component analysis (PCA) and analysis of variance (ANOVA). Using the *pca* function from the *scikit learn* python package (version 0.24.1; Virtanen et al. 2020), which uses a covariance matrix for the eigen decomposition, we reduced the 2100 dimensions of the reflectance data to the first 10 principal components (PC). PCA was applied spearately to different genotype groupings detailed below. For each of the first 10 PCs, we evaluated its ability to separate the genotypes using an ANOVA according to the methods in Cavender-Bares et al. (2016), followed by Tukey’s pairwise HSD tests. These methods were performed in python using the *statsmodels* package (version 0.12.2; Seabold and Perktold 2010) to compare genotypes separately for each of the following six groups: (1) Hawaii Island incana, glaberrima, newellii, and polymorpha; (2) glaberrima-incana, glaberrima, and Hawaii Island incana; (3) incana-polymorpha, Hawaii Island incana, and polymorpha; (4) newellii-polymorpha, newellii, and polymorpha; (5) glaberrima-polymorpha, glaberrima, and polymorpha; and (6) incana from Oahu and Hawaii Island (Table 1).

Although the PCA allowed us to determine if the spectra were differentiable, we used the spectral similarity index (SSI; Eq. 1; Somers et al. 2009, 2012, 2015) to quantify spectral overlap between varieties. The SSI calculates the spectral distance between populations *i* and *j* for each wavelength:

1 $$\text{SSI}= \frac{\left|{\overline{R} }_{b,i}-{\overline{R} }_{b,j}\right|}{sd\left({R}_{b,i}\right)+sd\left({R}_{b,j}\right)}$$

where *R* is the brightness-normalized reflectance for each group over *n* spectral bands. Rather than performing pairwise comparisons, population *j* was represented by pooled reflectance data from all varieties (including Oahu and Hawaii Island incana). In doing so, we estimated the degree to which each variety diverged spectrally from all varieties*.* SSI has been used to estimate species turnover (Somers et al. 2015) and as a means of determining which wavelengths distinguish classes (Asner et al. 2018). Here, we plotted SSI across the entire spectrum to quantify the degree of separation, with higher SSI values indicating a higher degree of spectral overlap, between spectra of the *M. polymorpha* varieties. Further, we calculated the mean SSI by taking an average of 1/SSI across all bands (Eq. 2).

2 $$\mathrm{mean SSI}= \frac{1}{n}\sum_{b=0}^{n}\frac{sd\left({R}_{b,i}\right)+sd\left({R}_{b,j}\right)}{\left|{\overline{R} }_{b,i}-{\overline{R} }_{b,j}\right|}$$

To understand within-variety variation, we calculated the mean spectra and coefficient of variation (CV) for each variety across the spectra. The CV is a standardized measure of variation that allows for visual comparison among samples across the full spectrum. Here, we use the CV to visually assess regions of the spectrum that show the greatest variation within each genotype of *M. polymorpha*. Although this investigation is useful for visualizing diversity in terms of reflectance between varieties, band-by-band assessments of CV are limited because spectra are derived from broader features related to chemical interactions with light.

Lastly, we examined variation among genotypes in leaf traits derived from the reflectance data. Leaf chemical traits were estimated from reflectance spectra using chemometric equations specific to *M. polymorpha* developed by Asner et al. (2018). These spectral−chemical relationships were determined using the partial least squares regression (PLSR) – prediction residual error sum of squares (PRESS) method that has been used to develop universal chemometric equations for broadleaf species (Asner et al. 2009, 2015; Asner and Martin 2008). As these methods approximate leaf traits, we use them as a means of comparing leaf traits between groups rather than interpreting their absolute value. We estimated eight chemical traits (Table SI 1) using the equations specific to *M. polymorpha*, including the photosynthetic pigments chlorophylls a and b, the structural molecules lignin and cellulose, and the secondary traits phenols and tannins. Chlorophylls a and b were summed and represented as chlorophyll a + b. Further, nonstructural carbohydrates (NSC) like sugars and starch were estimated along with total nitrogen (N) and total carbon (C). Leaf mass per unit area (LMA) was calculated using leaf area and dry weights quantified from the collected leaves, described above. When discussing leaf trait data, we refer to the chemical leaf traits estimated from the reflectance data as well as the LMA calculated from leaves. Significance of differences in leaf traits between genotypes in the groupings described above was quantified using ANOVA and Tukey HSD tests. All analyses were done using python version 3.6.9.

In summary, we first used principal component analysis (PCA) to reduce this highly dimensional dataset into fewer components that captured a larger proportion of the variance. We then determined whether any of the components could separate the varieties as well as F1 hybrids from their parent varieties using ANOVA and Tukey HSD. To understand differences in reflectance between and within the varieties, we used the spectral similarity index (SSI) and compared their coefficient of variation (CV) and leaf traits.

## Results

### Spectral divergence among varieties

PCAs of leaf spectra (Table 2) separated all varieties in pairwise comparison except glaberrima and incana. Two PCs (PC1 and PC5) derived from the reflectance spectra significantly differentiated the varieties (*p* = 0.003 for each; Table 2). In the pairwise comparison, incana and newellii were separable in both PC1 and PC5. PC1 additionally separated glaberrima and polymorpha as well as newellii and polymorpha. Glaberrima and newellii as well as incana and polymorpha were differentiable in PC5. The only taxon pair that was not separable in the first 10 PCs of the reflectance data was glaberrima and incana.

**Table 2:** Results showing the statistical separability of *M. polymorpha* varieties using spectra

ANOVA *p*-value	Genotype 1	Genotype 2	Mean difference	*P*-adj	Lower	Upper	Reject H_0_
Principal Component 1							
*p*-value = 0.003	Glaberrima	Incana	– 3.5	0.109	– 7.6	0.6	FALSE
	Glaberrima	Newellii	0.7	0.9	– 3.4	4.8	FALSE
	**Glaberrima**	**Polymorpha**	– **4.7**	**0.020**	– **8.8**	**– 0.6**	**TRUE**
	**Incana**	**Newellii**	**4.2**	**0.044**	**0.1**	**8.3**	**TRUE**
	Incana	Polymorpha	– 1.2	0.825	– 5.3	2.9	FALSE
	**Newellii**	**Polymorpha**	– **5.4**	**0.007**	– **9.5**	**– 1.3**	**TRUE**
Principal Component 5							
*p*-value = 0.003	Glaberrima	Incana	– 0.1	0.9	– 0.6	0.4	FALSE
	**Glaberrima**	**Newellii**	**0.6**	**0.018**	**0.1**	**1.1**	**TRUE**
	Glaberrima	Polymorpha	0.4	0.112	– 0.1	0.9	FALSE
	**Incana**	**Newellii**	**0.7**	**0.006**	**0.2**	**1.1**	**TRUE**
	**Incana**	**Polymorpha**	**0.5**	**0.043**	**0.0**	**1.0**	**TRUE**
	Newellii	Polymorpha	– 0.2	0.789	– 0.7	0.3	FALSE

Pairwise Tukey results of significant PC axes according to the ANOVA are displayed. ANOVA *p*-value is presented in column one. The genotypes being compared in the pairwise Tukey are listed in columns two and three. Following this, their mean difference, adjusted *p*-value (*P*-adj), and their lower and upper bounds are presented. The second to last column (Reject H_0_) indicates whether the null hypothesis that the two genotypes do not differ along the listed PC is rejected. Variety pairs differentiable according to Tukey’s tests are highlighted. See Fig. SI 3 for data plotted in PC space and PC loadings across VSWIR spectra

Genotype pairs that differed significantly are bolded

**Fig. 1:**
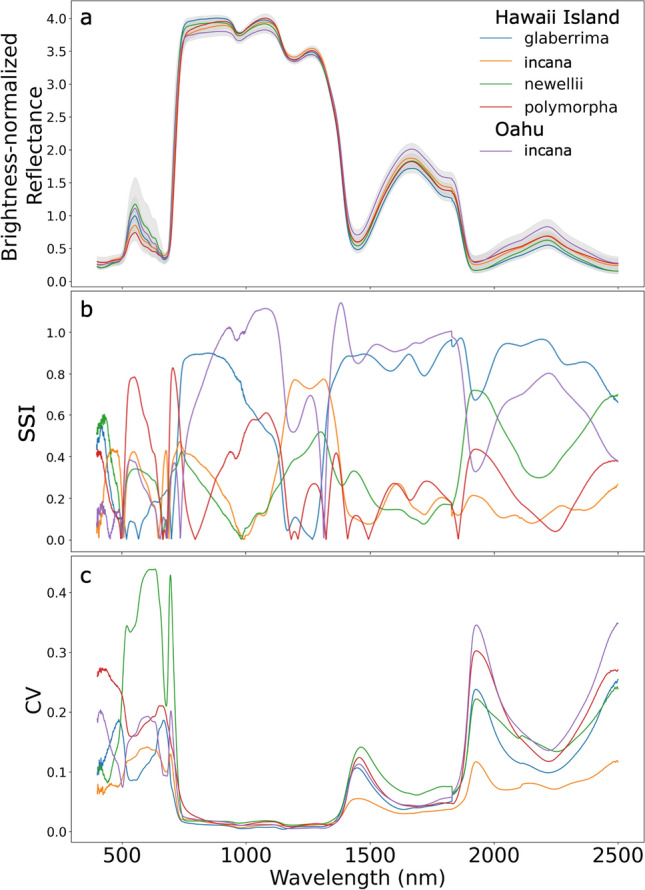
**a** Mean brightness-normalized reflectance (represented as a percentage) and **b** coefficient of variation (CV) of reflectance values for the four *M. polymorpha* Hawaii Island varieties and Oahu incana. See Fig. SI 2 for reflectance prior to brightness-normalization. **c** Spectral separability of all Hawaii Island and Oahu genotypes. Spectral separability was calculated for each wavelength (Eq. 1). Higher values indicate less spectral overlap. See Table 3 for average SSI values

When visually comparing spectra of the four Hawaii Island varieties, the mean brightness-normalized spectra vary most in the visible (400–700 nm) and shortwave-infrared (SWIR; 1500–2500 nm) wavelength regions (Fig. 1a). According to the spectral separability index (SSI), separation between the varieties occurred across the spectra (Fig. 1b), with polymorpha having the greatest mean SSI (29; Table 3). Both polymorpha and Hawaii Island incana were most distinct in the visible and parts of the infrared while glaberrima had the greatest separability in the SWIR and infrared (Fig. 1b). Glaberrima and incana were similar in their degree of spectral overlap with SSI values of 9 and 11, respectively (Table 3). Newellii, which had the highest separability after ~ 1800 nm, had the lowest mean SSI (7; Fig. 1b; Table 3).

**Table 3:** Within-variety spectral similarity of samples on Hawaii Island and Oahu

Island	Variety	SSI
Hawaii	Glaberrima	9
	Incana	11
	Newellii	7
	Polymorpha	29
Oahu	Incana	4

Results of mean spectral similarity index (SSI) calculated according to Eq. 2. For each spectral channel, the summed standard deviation was divided by the difference between means. These results were summed across the VSWIR spectra and divided by the total number of channels. SSI denotes spectral similarity of the mean spectra and spectral variance between the indicated variety and all varieties on Hawaii Island (and Oahu). Lower values indicate less spectral overlap

**Fig. 2:**
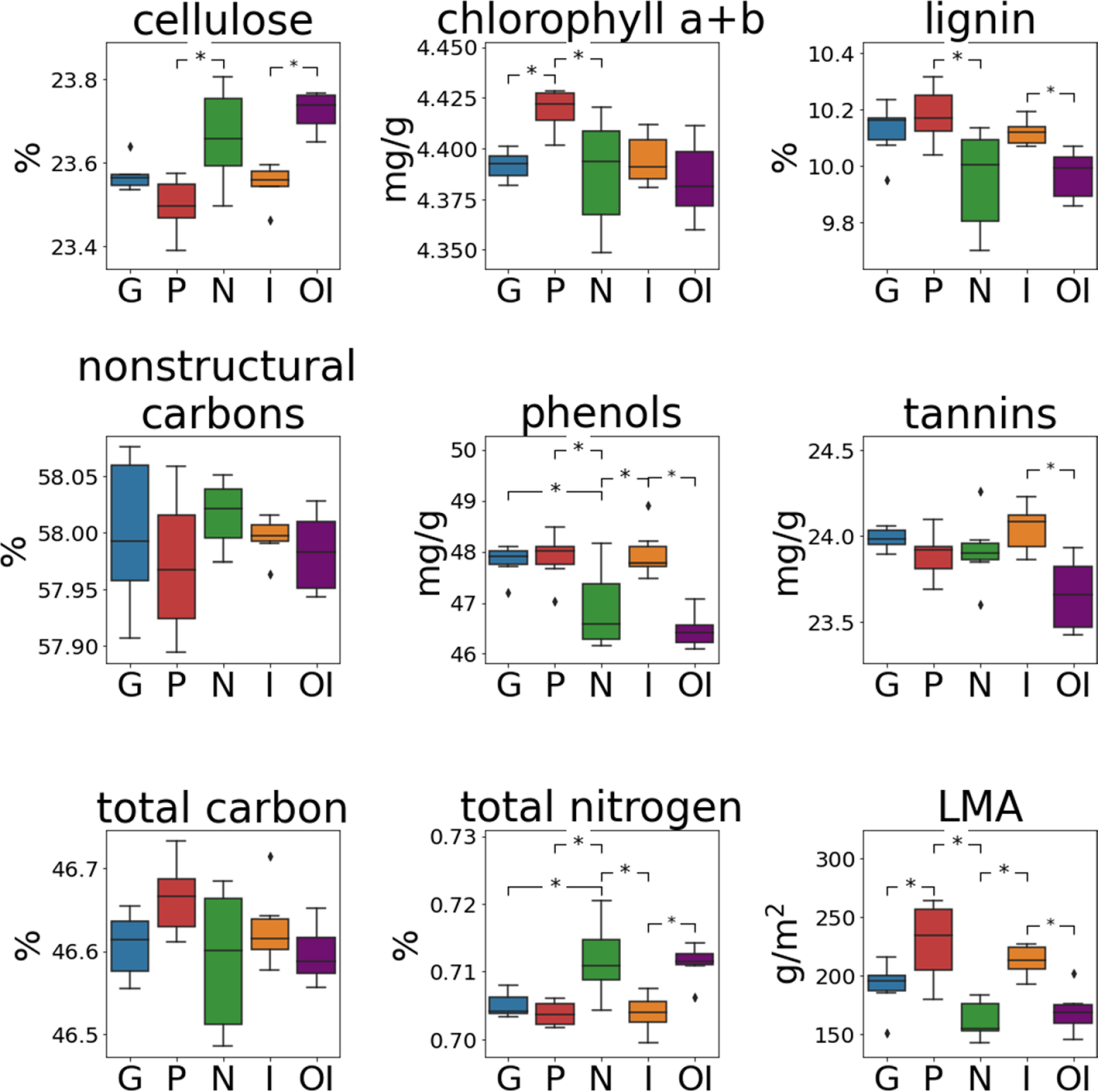
Boxplots of leaf traits for the Hawaii Island varieties glaberrima (G), polymorpha (P), newellii (N), and incana (I) as well as Oahu incana (OI). Hawaii Island varieties with traits that differed at a significance of *p* < 0.05 as determined by ANOVA and Tukey HSD are noted with an asterisk. Incana from Hawaii Island and Oahu were also compared using ANOVA, and significant differences are likewise noted with an asterisk. Boxplots denote quartile ranges, with the lower and upper bounds of the box indicating the 25th and 75th percentile. Middle lines in the box represent the median of the data, and the whiskers end at the group minimum and maximum. Outliers are shown as points

Among Hawaii Island varieties, incana had the least within-variety variation among the spectra, while newellii and polymorpha displayed the most variation according to the CV (Fig. 1c).Within-variety variation was greatest in the visible and SWIR regions of the spectrum (Fig. 1c). The CV of newellii peaked in the visible region, where newellii had not only the greatest within-variety variation but also the highest reflectance values. This result is also expressed in the estimated chemical data (Fig. 2), where newellii had a greater variability relative to the other varieties and lower values of chlorophyll a + b than polymorpha. While polymorpha likewise had a high CV in the visible, this variety had the lowest reflectance in this region compared to the other varieties, and this corresponded to high chlorophyll a + b. In the SWIR region, which is influenced by many leaf traits, within-variety variation was greatest for polymorpha and newellii, followed by glaberrima. Newellii had higher total N than all other varieties but lower LMA, total phenols, and lignin than some other varieties. Polymorpha had lower cellulose than newellii and higher LMA than glaberrima and newellii. Both polymorpha and glaberrima had a wide variation in NSC, and polymorpha had high variation in LMA. Incana had low variation in all the leaf traits except for tannins. Leaf traits were less useful than PCA for discriminating the varieties (Fig. 2). Cellulose, chlorophyll a + b, lignin, phenols, total N, and LMA separated newellii from all other varieties (Fig. 2). Beyond this, only polymorpha and glaberrima differed significantly in leaf traits (chlorophyll a + b and LMA; Fig. 2).

### Spectral patterns in hybrids

**Fig. 3:**
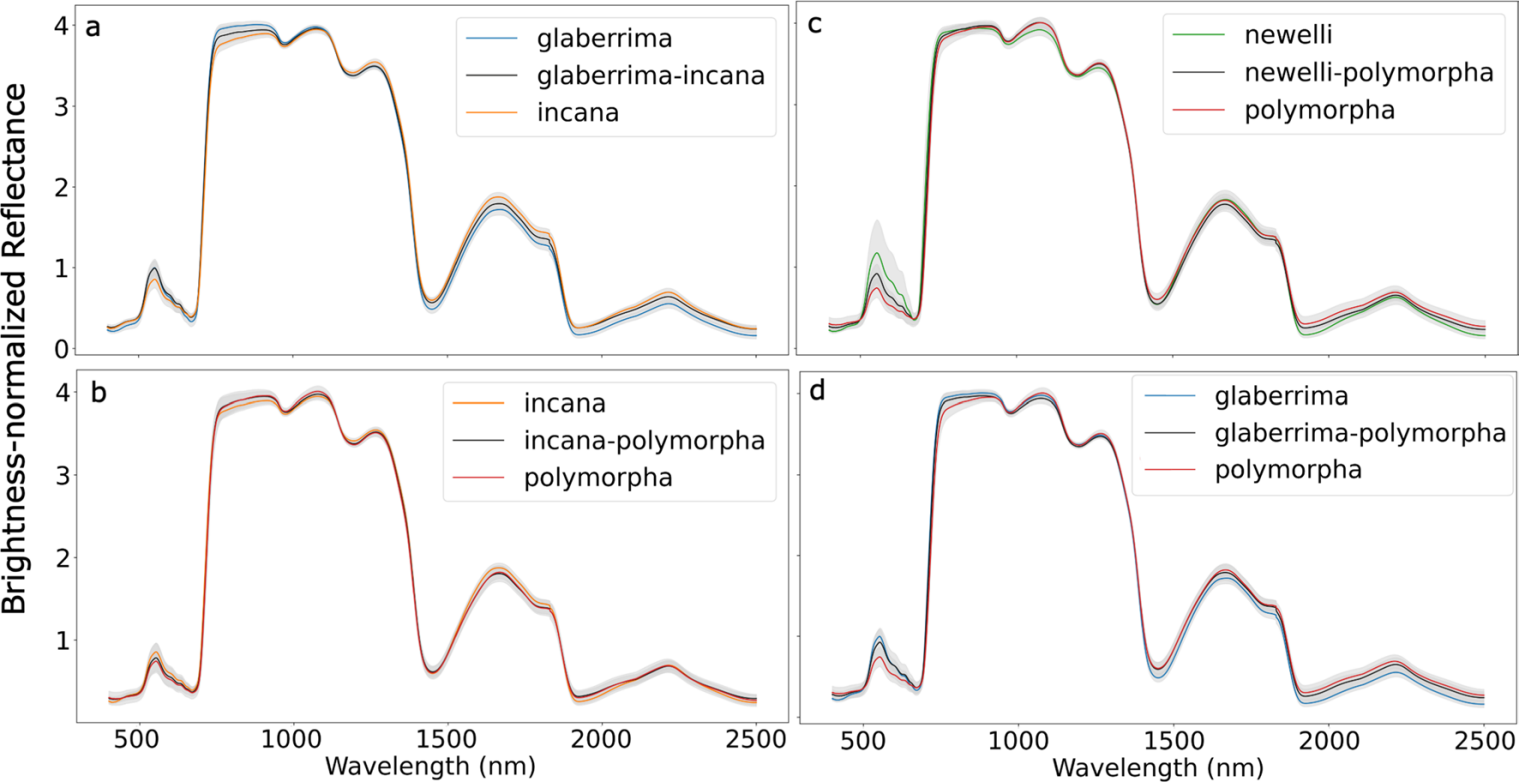
Mean brightness-normalized reflectance (represented as a percentage) of **a** the F1 hybrids glaberrima-incana (GI) and its parents, glaberrima (G) and incana (I); **b** the hybrid incana-polymorpha (IP) and its parents, incana (I) and polymorpha (P); the hybrid newellii-polymorpha (NP) and its parents, newellii (N) and polymorpha (P); **d** the hybrid glaberrima-polymorpha (GP) and its parents, glaberrima (G) and polymorpha (P). See supplementary information Figure SI 4 for CV of F1 hybrids

**Fig. 4:**
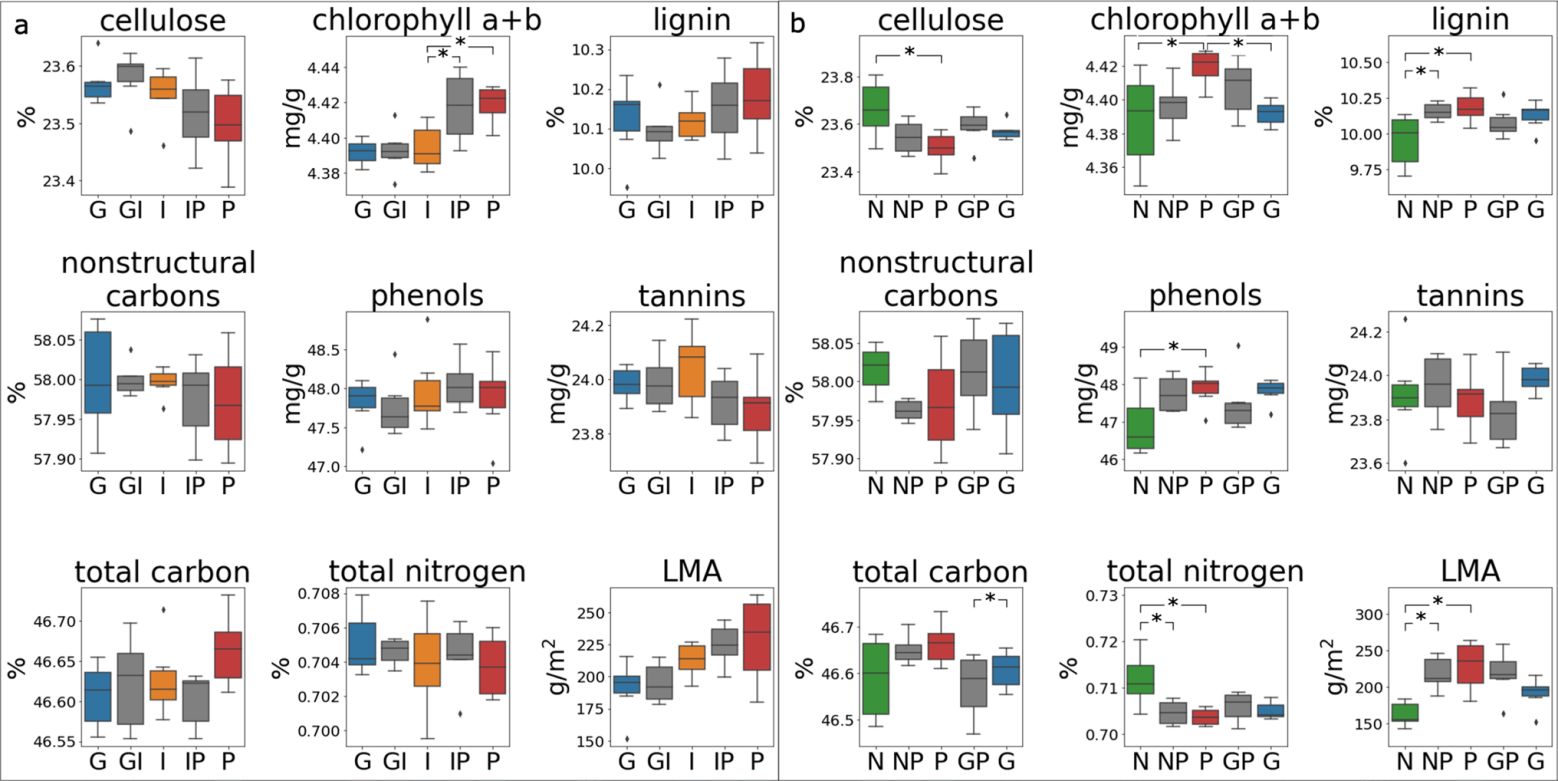
Boxplots of nine leaf traits for all F1 hybrids measured and their parents. The left figure **a** represents glaberrima (G), the F1 hybrid glaberrima-incana (GI), incana (I), the hybrid incana-polymorpha (IP), and polymorpha (P). The right figure **b** displays newellii (N), the hybrid newellii-polymorpha (NP), polymorpha (P), the hybrid glaberrima-polymorpha (GP), and glaberrima (G). Genotypes with traits that differed at a significance of *p* < 0.05 as determined by ANOVA and Tukey HSD are noted with an asterisk. Only groups of the F1 hybrid and their parents (Table 1) were compared using ANOVA and Tukey HSD. None of the traits differed significantly among GI, I, and G according to ANOVA

The four F1 hybrid genotypes demonstrated different patterns of leaf reflectance relative to their parental taxa. Spectral PC1 scores separated glaberrima and incana as well as glaberrima and glaberrima-incana hybrids (Table 4). Mean spectra of glaberrima-incana F1s fell between the mean spectra of their parent varieties but were closer to glaberrima in the visible and closer to incana between approximately 2000 and 2500 nm (Fig. 3a). Overall, the shape of the CV across the spectrum within glaberrima-incana F1s mirrored that of incana (Fig. SI 4a). None of the leaf traits differed between the glaberrima-incana F1s and either of their parent varieties (Fig. 4a). Variation in F1 leaf traits was often intermediate to or less than that of the parent varieties, except for total C and LMA (Fig. 4a).

**Table 4:** Results showing the statistical separation of *M. polymorpha* F1 hybrids and their parent varieties

ANOVA *p*-value	Genotype 1	Genotype 2	Mean difference	*P*-adj	Lower	Upper	Reject H_0_
Glaberrima-incana Principal Component 1							
*p*-value = 0.001	**Glaberrima**	**Glaberrima-incana**	– **2.9**	**0.047**	– **5.9**	**0.0**	**TRUE**
	**Glaberrima**	**Incana**	– **5.2**	**0.001**	– **8.1**	– **2.3**	**TRUE**
	Incana	Glaberrima-incana	– 2.2	0.150	– 5.1	0.7	FALSE
Incana-polymorpha Principal Component 3							
*p*-value = 0.03	**Incana**	**Incana-polymorpha**	**1.4**	**0.0363**	**0.1**	**2.7**	**TRUE**
	Incana	Polymorpha	1.2	0.0791	– 0.1	2.5	FALSE
	Polymorpha	Incana-polymorpha	– 0.2	0.9	– 1.5	1.1	FALSE
Newellii-polymorpha Principal Component 1							
*p*-value = 0.03	Newellii	Newellii-polymorpha	– 3.1	0.2101	– 7.7	1.4	FALSE
	**Newellii**	**Polymorpha**	– **5.4**	**0.0202**	– **10.0**	**– 0.8**	**TRUE**
	Polymorpha	Newellii-polymorpha	– 2.3	0.4263	– 6.9	2.3	FALSE
Glaberrima-polymorpha Principal Component 1							
*p*-value = 0.01	Glaberrima	Glaberrima-polymorpha	3.1	0.102	– 0.5	6.8	FALSE
	**Glaberrima**	**Polymorpha**	**4.9**	**0.01**	**1.2**	**8.5**	**TRUE**
	Polymorpha	Glaberrima-polymorpha	1.7	0.452	– 1.9	5.4	FALSE

Each grouping had only one significant principal component (PC) according to ANOVA. The results of the Tukey’s pairwise test for these PCs are shown. For each genotype pairing (columns two and three), their mean difference, adjusted *p*-value (*P*-adj), and lower and upper bounds are presented. The final column indicates whether the null hypothesis that the genotype pairings do not differ is rejected. See Fig. SI 5 for samples plotted in PC space

Genotype pairs that differed significantly are bolded

The incana-polymorpha F1 trees showed intermediate values for many of the leaf traits and within-genotype spectral variation, though their mean spectra most often mirrored those of polymorpha (Fig. 3b). Consistent with this trend, PC3 scores (but not PC1 or PC2 scores) separated incana-polymorpha F1s from incana but not polymorpha (Fig. 3b; Table 4). Similar to the glaberrima-incana F1s, spectral variation (CV) of incana-polymorpha most resembled that of incana in shape, but was often intermediate or closer to the other parent (here, polymorpha) in magnitude (Fig. SI 4b). Both incana-polymorpha F1s and polymorpha had higher chlorophyll a + b than incana (Fig. 4a). Many of the other leaf traits of the F1s displayed values intermediate to those of the parent values, though the variation of the hybrid data was often greater than that of either parent.

The mean spectral values of newellii-polymorpha F1s were intermediate in the visible, closely followed polymorpha in the infrared and beyond ~ 1700 nm, and were lower than either parent between 1500 and 1700 nm (Fig. 3c). Newellii and polymorpha was the only pair of genotypes in the newellii-polymorpha group that was separable by any PC scores (Table 4). Leaf trait data indicated that many of the F1 traits were intermediate to those of the parent varieties, but within-F1 variation was lower than variation within either parent for lignin, NSC, and total C (Fig. 4b). In contrast, tannin levels varied more among F1 trees than among trees of either parent. Four of the leaf traits separated newellii and polymorpha, while total N, lignin, and LMA separated newellii and newellii-polymorpha (Fig. 4b). Polymorpha and newellii-polymorpha F1s did not differ in any of the leaf traits.

Mean spectra of glaberrima-polymorpha F1s largely fell between those of the parent varieties, but more closely followed glaberrima in the visible and polymorpha in the SWIR (Fig. 3d). Only the parent varieties were differentiable using reflectance spectra (Table 4). Glaberrima-polymorpha F1s had lower total C relative to polymorpha, although within-group variation of total C was greater in the hybrid than either parent (Fig. 4b). LMA was the only leaf trait for which glaberrima-polymorpha F1s were intermediate to the parents in both median value and within-group variation. The glaberrima-polymorpha outlier values for tannins, lignin, and cellulose were taken from the same plant (Fig. 4b).

### Comparing populations across islands

Trees of incana from Oahu and Hawaii Island were compared with assess inter-island divergence of leaf spectra (Fig. 1). Across the full spectrum, except in the infrared (~ 750–1700 nm), mean spectral reflectance of incana was greater for trees from Oahu than those from Hawaii Island (Fig. 1a). The CV was similarly greater for Oahu trees across the spectra, and the shapes of the CV were similar only in the visible (Fig. 1c). PC1 scores significantly differentiated leaf spectra of incana from the different islands (*p* value < 0.05), and Oahu incana, with an SSI of 4, had the lowest SSI of all the varieties by nearly a factor of four (Fig. 1b; Table 3). Six of the leaf chemical traits differed between islands (Fig. 2). Oahu incana had higher cellulose and total N concentrations, but lower lignin, phenols, LMA, and tannins. Qualitative comparisons suggested that within-group trait variation was greater for Oahu incana in cellulose, lignin, NSC, and tannins.

## Discussion

We measured the leaf spectra of several genotypes of a landscape-dominant tree species and demonstrated separation of ecologically diverged varieties across the geographic scale of east Hawaii Island. Leaf reflectance data successfully distinguished all but one pair of varieties of *M. polymorpha* on Hawaii Island as well as populations of the same variety from different islands. Spectral reflectance measures from four classes of F1 hybrids led to less successful discrimination of intraspecific hybrids from their parental varieties, as expected. However, the results suggest that reflectance spectra should be useful for the detection of *M. polymorpha* hybrid zones using airborne imaging spectroscopy and that with increased sample size, discrimination of individual F1 hybrids from parental taxa may be possible.

### Spectral divergence among varieties

Biodiversity estimates based on imaging spectroscopy, in accordance with the spectral variability hypothesis, have been made across many landscapes (Féret and Asner 2011; Schäfer et al. 2016), but few studies have investigated how spectral variability captures intraspecific variation at finer scales (Cavender-Bares et al. 2016; Czyż et al. 2023; McManus et al. 2016). The current study demonstrates the potential of reflectance spectra to capture the genetic variation within a single hyperdominant tree species. Cavender-Bares et al. (2016) similarly demonstrated separability of common-garden *Quercus oleoides* from populations across Central America where gene flow was limited due to the geographic separation of populations. Here we demonstrate spectral differentiation at scales much smaller than several hundred kilometers as monodominant stands of different *M. polymorpha* variants can exist directly adjacent to one another – a promising first step toward landscape-scale mapping of this species. Further, the leaf reflectance and derived trait data may reflect the differential adaptation of the four varieties of *M. polymorpha* to contrasting environmental niches in accordance with the spectral variability hypothesis (Palmer et al. 2000, 2002), as is discussed below.

The spectral signatures of the four varieties of *M. polymorpha* on Hawaii Island were separable in pairwise comparisons, except for those of the two successional varieties, incana and glaberrima. Despite their distinct leaf phenotypes, pubescent incana and glabrous glaberrima are the most weakly genetically differentiated pair of varieties on Hawaii Island (DeBoer and Stacy 2013; Stacy et al. 2014) and Oahu (Stacy et al. 2020). Weak differentiation is consistent with their likely multi-million-year history of alternating periods of isolation by selection on new (incana) and old (glaberrima) lava flows and periods of hybridization on intermediate-aged flows (Corn and Hiesey 1973; Drake and Mueller-Dombois 1993; Kitayama et al. 1997; Stacy et al. 2017; Stacy and Sakishima 2019). Glaberrima and incana were not differentiable when all four varieties were included in the analysis; however, they were separable in the analysis comprising just these varieties and their hybrids. This result suggests that classifying glaberrima and incana using airborne imaging spectroscopy will be possible, but it may require the training of a secondary classification model on these varieties alone. In addition to their lack of separability via reflectance data, these varieties had a similar degree of spectral overlap (SSI) with the other varieties. Notably, incana had the lowest CV of reflectance of any variety. This low variation may be due to lower genetic variation among sampled incana due to purifying selection (Cvijović et al. 2018) in the harsh abiotic environments of new lava flows or due to the narrow sampling of incana for this study (i.e., from a single population) relative to the other varieties.

The spectral signatures and leaf traits recorded for polymorpha were consistent with values expected for high-elevation plants. Polymorpha dominates forests above ~ 1400 m and exhibits many traits associated with high-elevation plants, such as slow growth, compact form, and highly pubescent leaves (Dawson and Stemmermann 1990; Homeier et al. 2010; King et al. 2013; Yang et al. 2008). The high LMA observed in polymorpha compared to the other varieties is consistent with expectations, as thicker leaves are often associated with high-elevation plants (Read et al. 2014). Lignin is associated with tensile strength (Trupiano et al. 2012; Zhang et al. 2014), and high lignin in polymorpha may be an adaptation to the mechanical stress of wind at high elevations (Zaborowska et al. 2023). High LMA and chlorophyll a + b in polymorpha are consistent with the relatively higher total chlorophyll and lower leaf surface area observed in common-garden trees of *M. polymorpha* derived from high-elevation, open-pollinated seeds (Martin et al. 2007). High chlorophyll a + b in polymorpha may be related to leaf pubescence, as pubescent polymorpha leaves self-shade to reduce damage to photosystems (Martin et al. 2007). Low peak reflectance in the visible spectrum is supported by the high chlorophyll content and suggests that polymorpha captures more light than the other varieties (Martin et al. 2007).

The CV of the reflectance spectra was high for polymorpha, which was unexpected given the lower genetic variation of polymorpha relative to other varieties of *M. polymorpha* on Hawaii Island (Stacy et al. 2014). Moreover, despite its high genetic differentiation relative to incana and glaberrima (DeBoer and Stacy 2013), polymorpha had the greatest spectral overlap with these taxa. Although lower trait variability at high elevations has been observed using imaging spectroscopy data in Peru (Asner et al. 2016) and Hawaii (Seeley et al. 2023), polymorpha had high variability in its reflectance spectra. This appears to be due primarily to high variation in NSC and LMA, which may be a result of growing plants adapted to high elevations in low elevations or to sampling from young potted plants as opposed to full-grown trees. Comparisons of high- versus low-elevation *M. polymorpha *in situ revealed lower CV of canopy reflectance and trait variability at high elevations (Martin and Asner 2009; Seeley et al. 2023), supporting the conclusion that the greenhouse growing conditions affected polymorpha.

Reflectance spectra for newellii were generally consistent with isolation of small populations in separate riparian environments. Newellii is restricted to small, linear populations along riparian corridors on east Hawaii Island (Dawson and Stemmermann 1990; Ekar et al. 2019). The relatively strong genetic isolation of newellii from the other varieties (mean pairwise F_ST_ between newellii populations and populations of all other varieties = 0.13; max = 0.25; pairwise F_ST_ between glaberrima and polymorpha = [0.040, 0.137], incana and glaberrima = [0.029, 0.117], and incana and polymorpha = [0.051, 0.079] on young and old substrates, respectively; (Stacy et al. 2014) likely explains the low degree of spectral overlap observed in the SSI. Newellii populations are significantly diverged from each other due to genetic drift (Stacy et al. 2014), and individuals included in this study originated from different populations. Structural flexibility reduces drag in water and is a common adaptation in plants contending with flowing water (Dittrich et al. 2012). As lignin adds rigidity to foliage (dos Santos Abreu et al. 1999), the low lignin observed in leaves of newellii is consistent with adaptation of this variety to high river discharge events (Ekar et al. 2019) as well as the lignification suppression observed in the roots of flood-stressed soybeans (Komatsu et al. 2010). The relatively high total N in newellii leaves may indicate that newellii has higher protein concentration than the other varieties. While some riparian plants use specialized proteins to withstand flooding events (Xue et al. 2020), this has yet to be investigated in newellii. Further, reflectance of visible light was greatest for newellii, which may be a means of photoprotection. Newelli leaves, like those of glaberrima which had the second highest reflectance in the visible, are typically glabrous and therefore do not self-shade via pubescence.

### Spectral patterns in hybrids

Patterns of spectra and leaf traits varied across the four F1 genotypes. The glaberrima-incana, incana-polymorpha, and newellii-polymorpha F1s largely showed levels intermediate to those of the parent varieties, whereas the glaberrima-polymorpha F1s did not. Leaf traits of glaberrima-polymorpha often ranged higher or lower than those for either parent, although few of the differences were significant. Interestingly, these same patterns match those observed in the phenotypes of 2-year-old seedlings of these same four F1 genotypes, which were intermediate for all F1s except glaberrima-polymorpha (Stacy et al. 2016, unpub. data).

Phylogenetic signal in reflectance spectra has been demonstrated in multiple genera (Blonder et al. 2020; Cavender-Bares et al. 2016; Czyż et al. 2020; Madritch et al. 2014; McManus et al. 2016; Meireles et al. 2020). Here, we show inheritance patterns of reflectance spectra in intraspecific F1 hybrids of *M. polymorpha*. Through this study, we hope to understand the applicability of imaging spectroscopy in classifying hybrids in landscape-wide mapping efforts. Of the four F1 genotypes included in this study, only glaberrima-incana and incana-polymorpha were separable from one of the parent varieties using leaf spectra. In the case of glaberrima-incana, spectra could distinguish the hybrid from glaberrima, whereas individual leaf traits could not. For this hybrid and its parents, leaf traits were not distinct enough to discriminate the genotypes. For the other F1 genotypes, at least one of the leaf traits differed significantly between the hybrid and one parent variety. These results indicate that classifying hybrids using airborne imaging spectroscopy may be possible with an increased sample size but will likely require both PCA and leaf trait estimations from spectral data to capitalize on all the information present in the data.

### Comparing populations across islands

We assessed whether spectra of *M. polymorpha* var. *incana* from islands of differing ages are differentiable and consistent with their contrasting environments. As found in other studies of conspecific populations sampled across a broad spatial scale (Cavender-Bares et al. 2016; Madritch et al. 2014), we found that the populations of incana from Hawaii Island and Oahu had distinct spectral signatures. Further, the SSI indicated that Oahu incana were more distinct spectrally than any of the Hawaii varieties. These results are consistent with a higher genetic similarity of populations within islands than among islands (Choi et al. 2021; Percy et al. 2008; Stacy and Sakishima 2019). Although the spectra of Oahu and Hawaii Island incana are separable, they share a characteristic shape in the CV between 500 and 750 nm. This shape was also present in all incana hybrids and includes a rounded peak around the red wavelengths as well as a sharp peak at the red edge. As the red edge defines the inflection point between red and infrared and has been linked to chlorophyll content, mesophyll structure, and leaf water content (Collins 1978; Horler et al. 1983), it is likely that variability patterns for one or all of these traits are present in incana and inherited by incana hybrids. Within-variety variation of leaf spectra was greater for Oahu incana, which may be due to the weaker purifying selection there relative to that on new lava flows on volcanically active Hawaii Island, the relatively narrow sampling of Hawaii Island incana in this study, or simply the older age of Oahu. Oahu, being approximately 3 million years older than Hawaii Island, has more available nitrogen in its soils (Vitousek et al. 1997), which results in greater trait variability (Asner et al. 2016; Ordoñez et al. 2009) and therefore spectral variability (Seeley et al. 2023).

## Conclusion

Using the highly variable, landscape-dominant tree species, *M. polymorpha*, grown in a common garden on Hawaii Island, we used leaf reflectance spectra and derived leaf traits to distinguish four ecologically diverged varieties and their hybrids with varying degrees of success. Further, we discussed the possible associations between the reflectance spectra and leaf trait data with local adaptation of the four varieties to their respective environments. The intersection of genetic analyses and geographical information systems (GIS) has been important in informing biogeographical research and conservation decisions that seek to protect genetic diversity (Koskela et al. 2013; Zonneveld et al. 2012); however, spatial genetic data of forests are limited. This study demonstrates that reflectance spectra can discriminate genotypes of *M. polymorpha,* suggesting that while the varieties and hybrids can be spatially mapped using airborne imaging spectroscopy, further investigations are necessary to determine if the resolution from canopy-level data will be stronger or weaker relative to leaf-level data (Jacquemoud et al. 2009; Jacquemoud and Baret 1990). As we plan for the use of imaging spectroscopy in biodiversity studies, the *M. polymorpha* model system will help us incorporate genetic variation rather than land-cover or morpho-taxonomic variation and pattern into conservation science and management.

## Supplementary Information

Below is the link to the electronic supplementary material.

Supplementary file1 (DOCX 4434 KB)

## Data Availability

The datasets generated during and/or analysed during the current study are available in the Figshare repository at 10.6084/m9.figshare.22714972.v1.

## References

[CR1] Aravanopoulos FA, Zsuffa L (1998). Heterozygosity and biomass production in *Salix*
*eriocephala*. Heredity.

[CR2] Arcade A, Faivre-Rampant P, Le Guerroué B, Pâques LE, Prat D (1996). Heterozygosity and hybrid performance in larch. Theor Appl Genet.

[CR3] Asner GP, Martin RE (2008). Spectral and chemical analysis of tropical forests: scaling from leaf to canopy levels. Remote Sens Environ.

[CR4] Asner GP, Martin RE, Ford AJ, Metcalfe DJ, Liddell MJ (2009). Leaf chemical and spectral diversity in Australian tropical forests. Ecol Appl.

[CR5] Asner GP, Martin RE, Anderson CB, Knapp DE (2015). Quantifying forest canopy traits: imaging spectroscopy versus field survey. Remote Sens Environ.

[CR6] Asner GP, Knapp DE, Anderson CB, Martin RE, Vaughn N (2016). Large-scale climatic and geophysical controls on the leaf economics spectrum. Proc Natl Acad Sci.

[CR7] Asner GP, Martin RE, Knapp DE, Tupayachi R, Anderson CB, Sinca F, Vaughn NR, Llactayo W (2017). Airborne laser-guided imaging spectroscopy to map forest trait diversity and guide conservation. Science.

[CR8] Asner GP, Martin RE, Keith LM, Heller WP, Hughes MA, Vaughn NR, Hughes RF, Balzotti C (2018). A spectral mapping signature for the rapid ohia death (ROD) pathogen in Hawaiian Forests. Remote Sens.

[CR9] Bergmann F, Hosius B (1996). Effects of heavy metal polluted soils on the genetic structure of Norway spruce seedling populations. Water Air Soil Pollut.

[CR11] Blonder B, Graae BJ, Greer B, Haagsma M, Helsen K, Kapás RE, Pai H, Rieksta J, Sapena D, Still CJ, Strimbeck R (2020). Remote sensing of ploidy level in quaking aspen (Populus tremuloides Michx). J Ecol.

[CR12] Bourgaud F, Gravot A, Milesi S, Gontier E (2001). Production of plant secondary metabolites: a historical perspective. Plant Sci.

[CR13] Cavender-Bares J, Meireles JE, Couture JJ, Kaproth MA, Kingdon CC, Singh A, Serbin SP, Center A, Zuniga E, Pilz G, Townsend PA (2016). Associations of leaf spectra with genetic and phylogenetic variation in oaks: prospects for remote detection of biodiversity. Remote Sens.

[CR14] Choi JY, Dai X, Alam O, Peng JZ, Rughani P, Hickey S, Harrington E, Juul S, Ayroles JF, Purugganan MD, Stacy EA (2021). Ancestral polymorphisms shape the adaptive radiation of Metrosideros across the Hawaiian Islands. Proc Natl Acad Sci.

[CR15] Collins W (1978). Remote sensing of crop type and maturity. Photogramm Eng Remote Sens.

[CR16] Corn CA, Hiesey WM (1973). Altitudinal variation in Hawaiian Metrosideros. Am J Bot.

[CR17] Corn CA (1979) Variation in Hawaiian Metrosideros. [Ph.D., University of Hawai’i at Manoa]. https://www.proquest.com/docview/302913798/citation/51D6E6521A78400DPQ/1

[CR18] Crutsinger GM, Souza L, Sanders NJ (2008). Intraspecific diversity and dominant genotypes resist plant invasions. Ecol Lett.

[CR19] Cvijović I, Good BH, Desai MM (2018). The effect of strong purifying selection on genetic diversity. Genetics.

[CR20] Czyż EA, Guillén Escribà C, Wulf H, Tedder A, Schuman MC, Schneider FD, Schaepman ME (2020). Intraspecific genetic variation of a *Fagus*
*sylvatica* population in a temperate forest derived from airborne imaging spectroscopy time series. Ecol Evol.

[CR21] Czyż EA, Schmid B, Hueni A, Eppinga MB, Schuman MC, Schneider FD, Guillén-Escribà C, Schaepman ME (2023). Genetic constraints on temporal variation of airborne reflectance spectra and their uncertainties over a temperate forest. Remote Sens Environ.

[CR22] Dawson, J., & Stemmermann, L. (1990). Metrosideros (Gaud). In manual of the flowering plants of Hawai’i (pp. 964–970). Univ. Hawai’i Press

[CR23] Deacon NJ, Grossman JJ, Schweiger AK, Armour I, Cavender-Bares J (2017). Genetic, morphological, and spectral characterization of relictual Niobrara River hybrid aspens (Populus × smithii). Am J Bot.

[CR24] DeBoer N, Stacy EA (2013). Divergence within and among 3 varieties of the endemic tree, ‘Ōhi’a Lehua (*Metrosideros*
*polymorpha*) on the Eastern slope of Hawai’i Island. J Hered.

[CR25] Dittrich A, Aberle J, Schoneboom T (2012). Drag forces and flow resistance of flexible riparian vegetation. In Environmental fluid mechanics. CRC Press

[CR26] dos Santos Abreu H, Nascimento AM, Maria MA (1999). Lignin structure and wood properties. Wood Fiber Sci.

[CR27] Drake DR, Mueller-Dombois D (1993). Population development of rain forest trees on a chronosequence of Hawaiian lava flows. Ecology.

[CR28] Dupuis JR, Pillon Y, Sakishima T, Gemmill CEC, Chamala S, Barbazuk WB, Geib SM, Stacy EA (2019). Targeted amplicon sequencing of 40 nuclear genes supports a single introduction and rapid radiation of Hawaiian Metrosideros (Myrtaceae). Plant Syst Evol.

[CR29] Ekar JM, Price DK, Johnson MA, Stacy EA (2019). Varieties of the highly dispersible and hypervariable tree, *Metrosideros*
*polymorpha*, differ in response to mechanical stress and light across a sharp ecotone. Am J Bot.

[CR30] Féret J-B, Asner GP (2011). Spectroscopic classification of tropical forest species using radiative transfer modeling. Remote Sens Environ.

[CR31] Hallgren P, Ikonen A, Hjältén J, Roininen H (2003). Inheritance patterns of phenolics in F1, F2, and back-cross hybrids of willows: implications for herbivore responses to hybrid plants. J Chem Ecol.

[CR32] Homeier J, Breckle S-W, Günter S, Rollenbeck RT, Leuschner C (2010). Tree diversity, forest structure and productivity along altitudinal and topographical gradients in a species-rich Ecuadorian Montane rain forest. Biotropica.

[CR33] Horler DNH, Dockray M, Barber J (1983). The red edge of plant leaf reflectance. Int J Remote Sens.

[CR34] Hueni A, Bialek A (2017). Cause, effect, and correction of field spectroradiometer interchannel radiometric steps. IEEE J Sel Top Appl Earth Obs Remote Sens.

[CR35] Izuno A, Onoda Y, Amada G, Kobayashi K, Mukai M, Isagi Y, Shimizu KK (2022). Demography and selection analysis of the incipient adaptive radiation of a Hawaiian woody species. PLoS Genet.

[CR36] Jacquemoud S, Baret F (1990). PROSPECT: a model of leaf optical properties spectra. Remote Sens Environ.

[CR37] Jacquemoud S, Verhoef W, Baret F, Bacour C, Zarco-Tejada PJ, Asner GP, François C, Ustin SL (2009). PROSPECT+SAIL models: a review of use for vegetation characterization. Remote Sens Environ.

[CR38] Jelinski DE (1993). Associations between environmental heterogeneity, heterozygosity, and growth rates of populus tremuloides in a cordilleran landscape. Arct Alp Res.

[CR39] Johnson MTJ, Lajeunesse MJ, Agrawal AA (2006). Additive and interactive effects of plant genotypic diversity on arthropod communities and plant fitness. Ecol Lett.

[CR40] King GM, Gugerli F, Fonti P, Frank DC (2013). Tree growth response along an elevational gradient: climate or genetics?. Oecologia.

[CR41] Kitayama K, Pattison R, Cordell S, Webb D, Mueller-dombois D (1997). Ecological and genetic implications of foliar polymorphism inMetrosideros polymorphagaud (Myrtaceae) in a habitat matrix on Mauna Loa, Hawaii. Ann Bot.

[CR42] Knowles P, Grant MC (1981). Genetic patterns associated with growth variability in ponderosa pine. Am J Bot.

[CR43] Komatsu S, Kobayashi Y, Nishizawa K, Nanjo Y, Furukawa K (2010). Comparative proteomics analysis of differentially expressed proteins in soybean cell wall during flooding stress. Amino Acids.

[CR44] Koskela J, Lefèvre F, Schueler S, Kraigher H, Olrik DC, Hubert J, Longauer R, Bozzano M, Yrjänä L, Alizoti P, Rotach P, Vietto L, Bordács S, Myking T, Eysteinsson T, Souvannavong O, Fady B, De Cuyper B, Heinze B, Ditlevsen B (2013). Translating conservation genetics into management: pan-European minimum requirements for dynamic conservation units of forest tree genetic diversity. Biol Conserv.

[CR45] Kruse FA, Heidebrecht KB, Shapiro AT, Barloon PJ, Goetz AFH (1993). The spectral image processing system (SIPS) interactive visualization and analysis of imaging spectrometer data. Remote Sens Environ.

[CR10] Le Blanc D. (2015). Global sustainable development report 2015.

[CR46] Madritch MD, Kingdon CC, Singh A, Mock KE, Lindroth RL, Townsend PA (2014). Imaging spectroscopy links aspen genotype with below-ground processes at landscape scales. Phil Trans R Soc b: Biol Sci..

[CR47] Marron N, Ceulemans R (2006). Genetic variation of leaf traits related to productivity in a Populus deltoides × Populus nigra family. Can J for Res.

[CR48] Martin RE, Asner GP (2009). Leaf chemical and optical properties of metrosideros polymorpha across environmental gradients in Hawaii. Biotropica.

[CR49] Martin RE, Asner GP, Sack L (2007). Genetic variation in leaf pigment, optical and photosynthetic function among diverse phenotypes of *Metrosideros*
*polymorpha* grown in a common garden. Oecologia.

[CR50] McManus KM, Asner GP, Martin RE, Dexter KG, Kress WJ, Field CB (2016). Phylogenetic Structure of foliar spectral traits in tropical forest canopies. Remote Sens.

[CR51] Meireles JE, Cavender-Bares J, Townsend PA, Ustin S, Gamon JA, Schweiger AK, Schaepman ME, Asner GP, Martin RE, Singh A, Schrodt F, Chlus A, O’Meara BC (2020). Leaf reflectance spectra capture the evolutionary history of seed plants. New Phytol.

[CR52] Mitton JB, Knowles P, Sturgeon KB, Linhart YB, & Davis M (1981). Associations between heterozygosity and growth rate variables in three Western forest trees. 8.

[CR53] Morrison KR, Stacy EA (2014). Intraspecific divergence and evolution of a life-history trade-off along a successional gradient in Hawaii’s *Metrosideros*
*polymorpha*. J Evol Biol.

[CR54] Müller-Starck G (1985). Genetic differences between" tolerant" and" sensitive" beeches (*Fagus*
*sylvatica* L.) in an environmentally stressed adult forest stand. Silvae Genetica.

[CR55] Myneni RB, Ross J, Asrar G (1989). A review on the theory of photon transport in leaf canopies. Agric Meteorol.

[CR56] Oleksyn J, Prus-Glowacki W, Giertych M, Reich PB (1994). Relation between genetic diversity and pollution impact in a 1912 experiment with East European Pinussylvestris provenances. Can J for Res.

[CR57] Ordoñez JC, Van Bodegom PM, Witte J-PM, Wright IJ, Reich PB, Aerts R (2009). A global study of relationships between leaf traits, climate and soil measures of nutrient fertility. Glob Ecol Biogeogr.

[CR58] Orians CM, Griffiths ME, Roche BM, Fritz RS (2000). Phenolic glycosides and condensed tannins in Salix sericea, S. eriocephala and their F1 hybrids: not all hybrids are created equal. Biochem Syst Ecol.

[CR59] Palmer MW, Earls PG, Hoagland BW, White PS, Wohlgemuth T (2002). Quantitative tools for perfecting species lists. Environmetrics.

[CR60] Palmer MW, Wohlgemuth T, Earls P, Arevalo JR, Thompson S (2000). Opportunities for long-term ecological research at the Tallgrass Prairie Preserve, Oklahoma. Proceedings of the ILTER regional workshop: cooperation in long term ecological research in Central and Eastern Europe, Budapest, Hungary, 22.

[CR61] Percy DM, Garver AM, Wagner WL, James HF, Cunningham CW, Miller SE, Fleischer RC (2008). Progressive island colonization and ancient origin of Hawaiian *Metrosideros* (Myrtaceae). Proc R Soc b: Biol Sci.

[CR62] Read QD, Moorhead LC, Swenson NG, Bailey JK, Sanders NJ (2014). Convergent effects of elevation on functional leaf traits within and among species. Funct Ecol.

[CR63] Rhoades AM (2012). The evolution of reproductive barriers within an endemic Hawaiian tree species (Metrosideros polymorpha) across environmental extremes [M.S., University of Hawai’i at Hilo]. https://www.proquest.com/docview/1269522622/abstract/9FA75DDFEF6B4830PQ/1

[CR64] Schaberg PG, DeHayes DH, Hawley GJ, Nijensohn SE (2008). Anthropogenic alterations of genetic diversity within tree populations: Implications for forest ecosystem resilience. For Ecol Manage.

[CR65] Schäfer E, Heiskanen J, Heikinheimo V, Pellikka P (2016). Mapping tree species diversity of a tropical montane forest by unsupervised clustering of airborne imaging spectroscopy data. Ecol Ind.

[CR66] Seabold S, Perktold J. (2010). statsmodels: econometric and statistical modeling with python. 9th Python in Science Conference.

[CR68] Seeley MM, Martin RE, Vaughn NR, Thompson DR, Dai J, Asner GP (2023). Quantifying the variation in reflectance spectra of *Metrosideros polymorpha* canopies across environmental gradients. Remote Sens.

[CR69] Somers B, Delalieux S, Stuckens J, Verstraeten WW, Coppin P (2009). A weighted linear spectral mixture analysis approach to address endmember variability in agricultural production systems. Int J Remote Sens.

[CR70] Somers B, Zortea M, Plaza A, Asner GP (2012). Automated extraction of image-based endmember bundles for improved spectral unmixing. IEEE J Sel Top Appl Earth Obs Remote Sens.

[CR71] Somers B, Asner GP, Martin RE, Anderson CB, Knapp DE, Wright SJ, Van De Kerchove R (2015). Mesoscale assessment of changes in tropical tree species richness across a bioclimatic gradient in Panama using airborne imaging spectroscopy. Remote Sens Environ.

[CR72] Stacy EA, Sakishima T (2019). Phylogeography of the highly dispersible landscape-dominant woody species complex, metrosideros. Hawaii J Biogeogr.

[CR73] Stacy EA, Johansen JB, Sakishima T, Price DK, Pillon Y (2014). Incipient radiation within the dominant Hawaiian tree *Metrosideros*
*polymorpha*. Heredity.

[CR74] Stacy EA, Johansen JB, Sakishima T, Price DK (2016). Genetic analysis of an ephemeral intraspecific hybrid zone in the hypervariable tree, *Metrosideros*
*polymorpha*, on Hawai‘i island. Heredity.

[CR75] Stacy EA, Paritosh B, Johnson MA, Price DK (2017). Incipient ecological speciation between successional varieties of a dominant tree involves intrinsic postzygotic isolating barriers. Ecol Evol.

[CR76] Stacy EA, Sakishima T, Tharp H, Snow N (2020). Isolation of Metrosideros (`Ohi`a) Taxa on O`ahu increases with elevation and extreme environments. J Hered.

[CR77] Stacy EA, Ekar JM, Sakishima T. (n.d.). [Metrosideros polymorpha controlled crossess on Oahu and Hawaii Island] [Unpublished data].

[CR78] Stasinski L, White DM, Nelson PR, Ree RH, Meireles JE (2021). Reading light: leaf spectra capture fine-scale diversity of closely related, hybridizing arctic shrubs. New Phytol.

[CR79] Stemmermann L, Ihsle T (1993). Replacement of Metrosideros polymorpha, `Ohi`a Hawaiian dry forest succession. Biotropica.

[CR80] Tang T, Zhang N, Bongers FJ, Staab M, Schuldt A, Fornoff F, Lin H, Cavender-Bares J, Hipp AL, Li S, Liang Y, Han B, Klein A-M, Bruelheide H, Durka W, Schmid B, Ma K, Liu X (2022). Tree species and genetic diversity increase productivity via functional diversity and trophic feedbacks. Elife.

[CR81] Treseder KK, Vitousek PM (2001). Effects of soil nutrient availability on investment in acquisition of N and P in Hawaiian rain forests. Ecology.

[CR82] Trupiano D, Di Iorio A, Montagnoli A, Lasserre B, Rocco M, Grosso A, Scaloni A, Marra M, Chiatante D, Scippa GS (2012). Involvement of lignin and hormones in the response of woody poplar taproots to mechanical stress. Physiol Plant.

[CR83] van Zonneveld M, Scheldeman X, Escribano P, Viruel MA, Damme PV, Garcia W, Tapia C, Romero J, Sigueñas M, Hormaza JI (2012). Mapping genetic diversity of cherimoya (annona cherimola mill.): application of spatial analysis for conservation and use of plant genetic resources. PLoS ONE.

[CR84] Virtanen P, Gommers R, Oliphant TE, Haberland M, Reddy T, Cournapeau D, Burovski E, Peterson P, Weckesser W, Bright J, van der Walt SJ, Brett M, Wilson J, Millman KJ, Mayorov N, Nelson ARJ, Jones E, Kern R, Larson E, Vázquez-Baeza Y (2020). SciPy 1.0: fundamental algorithms for scientific computing in python. Nat Methods.

[CR85] Vitousek PM, Chadwick O, Crews TE, Fownes J, Hendricks DM, Herbert D (1997). Soil and ecosystem development across the Hawaiian Islands. GSA Today.

[CR86] Walters M, Scholes RJ (2017). The GEO handbook on biodiversity observation networks.

[CR87] Wright SD, Yong CG, Dawson JW, Whittaker DJ, Gardener RC (2000). Riding the ice age El Niño? Pacific biogeography and evolution of metrosideros subg. Metrosideros (Myrtaceae) inferred from nuclear ribosomal DNA. PNAS.

[CR88] Wright IJ, Reich PB, Westoby M, Ackerly DD, Baruch Z, Bongers F, Cavender-Bares J, Chapin T, Cornelissen JHC, Diemer M, Flexas J, Garnier E, Groom PK, Gulias J, Hikosaka K, Lamont BB, Lee T, Lee W, Lusk C, Villar R (2004). The worldwide leaf economics spectrum. Nature.

[CR89] Xue Y, Gao Y, Liu C, Liu S (2020). A styrene antioxidant NFA from riparian endophytic fungi enhances flooding tolerance in Arabidopsis. J Plant Interact.

[CR90] Yang Y, Körner C, Sun H (2008). The ecological significance of pubescence in saussurea medusa, a high-elevation Himalayan “Woolly Plant”. Arct Antarct Alp Res.

[CR91] Zaborowska J, Perry A, Cavers S, Wachowiak WM (2023). Evolutionary targets of gene expression divergence in a complex of closely related pine species. J Syst Evol.

[CR92] Zhang C-B, Chen L-H, Jiang J (2014). Why fine tree roots are stronger than thicker roots: the role of cellulose and lignin in relation to slope stability. Geomorphology.

