# Supplementary material for: Foliar functional and genetic variation in a keystone Hawaiian tree species estimated through spectroscopy
Source: Oecologia. 2023 May 12;202(1):15–28. doi: 10.1007/s00442-023-05374-1 (PMC10229453; doi:10.1007/s00442-023-05374-1)

**Foliar functional and genetic variation in a keystone Hawaiian tree species estimated through spectroscopy**

Seeley, M.^1,2^, Stacy, E.^3^, Martin, R.E.^1,2^, & Asner, G.P.^1,2^

**Supplementary Information**

**Table SI 1:** The eight leaf chemical traits estimated from reflectance spectra according to Asner et al., (2018).

| **Chemistry** | **Abbreviation** | **Units** |
| --- | --- | --- |
| Total Nitrogen | N | % |
| Phenols | Phenols | mg g^-1^ |
| Chlorophyll a+b | Chl | mg g^-1^ |
| Cellulose | Cellulose | % |
| Tannins | Tannins | mg g^-1^ |
| Lignin | Lignin | % |
| Total Carbon | C | % |
| Nonstructural Carbohydrates | NSC | % |

**
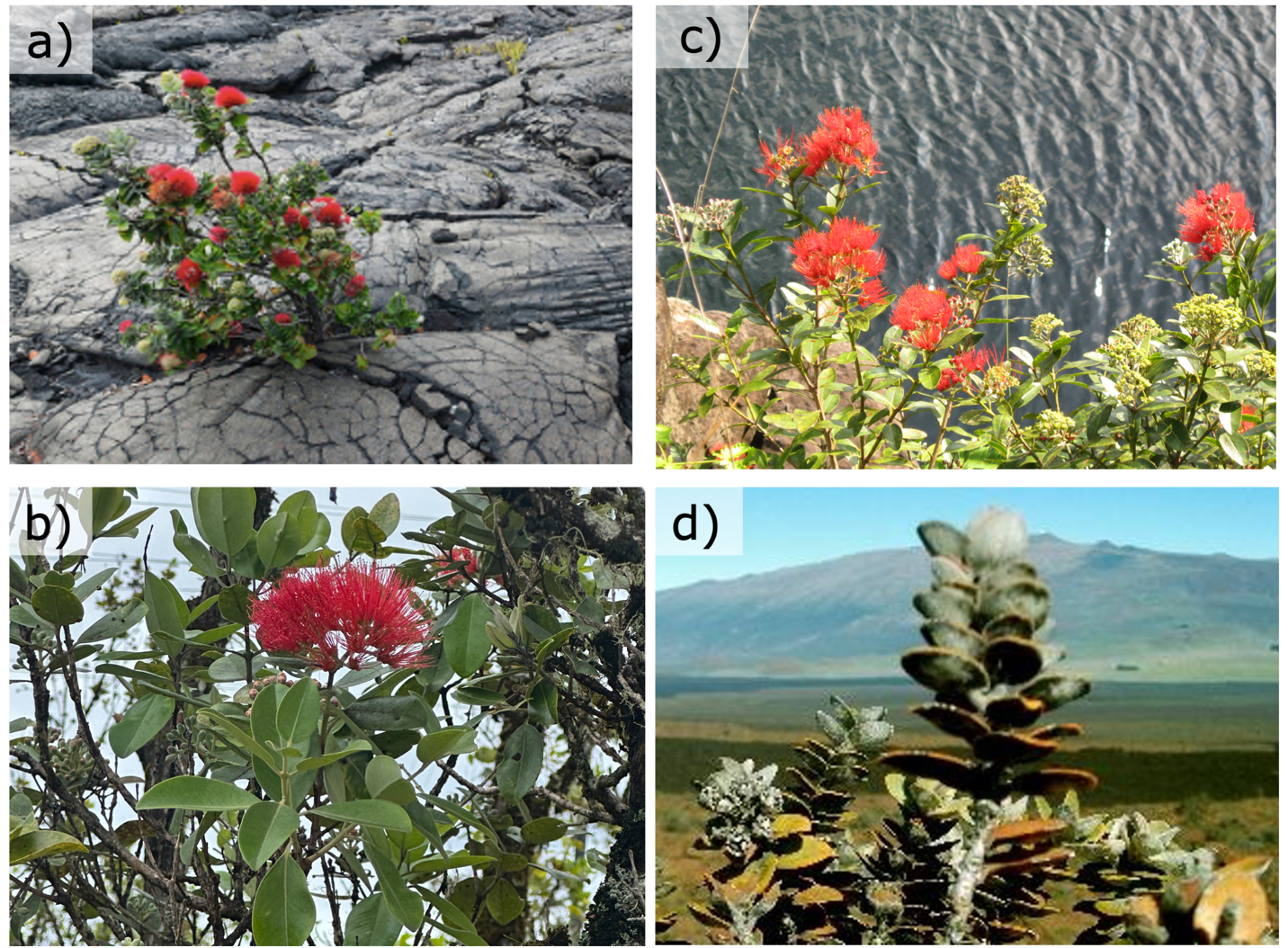
**

**Figure SI 1:** The four varieties of *Metrosideros polymorpha* on Hawaii Island*.* a) *M. polymorpha* var. *incana*. b) *M. polymorpha* var. *glaberrima.* c) *M. polymorpha* var. *newellii*. d) *M. polymorpha* var. *polymorpha.*

**
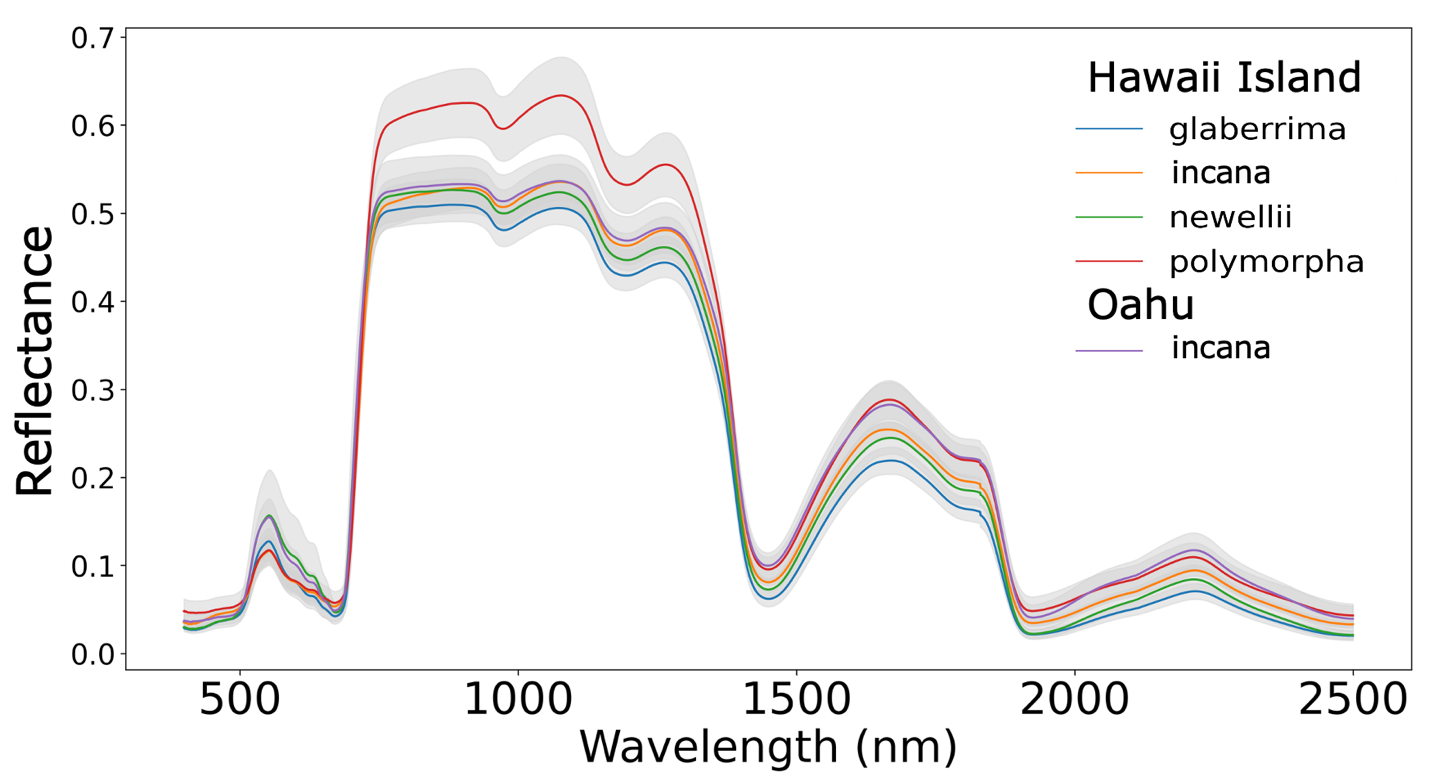
**

**Figure SI 2:** Reflectance of the four Hawaii Island varieties before brightness-normalization.


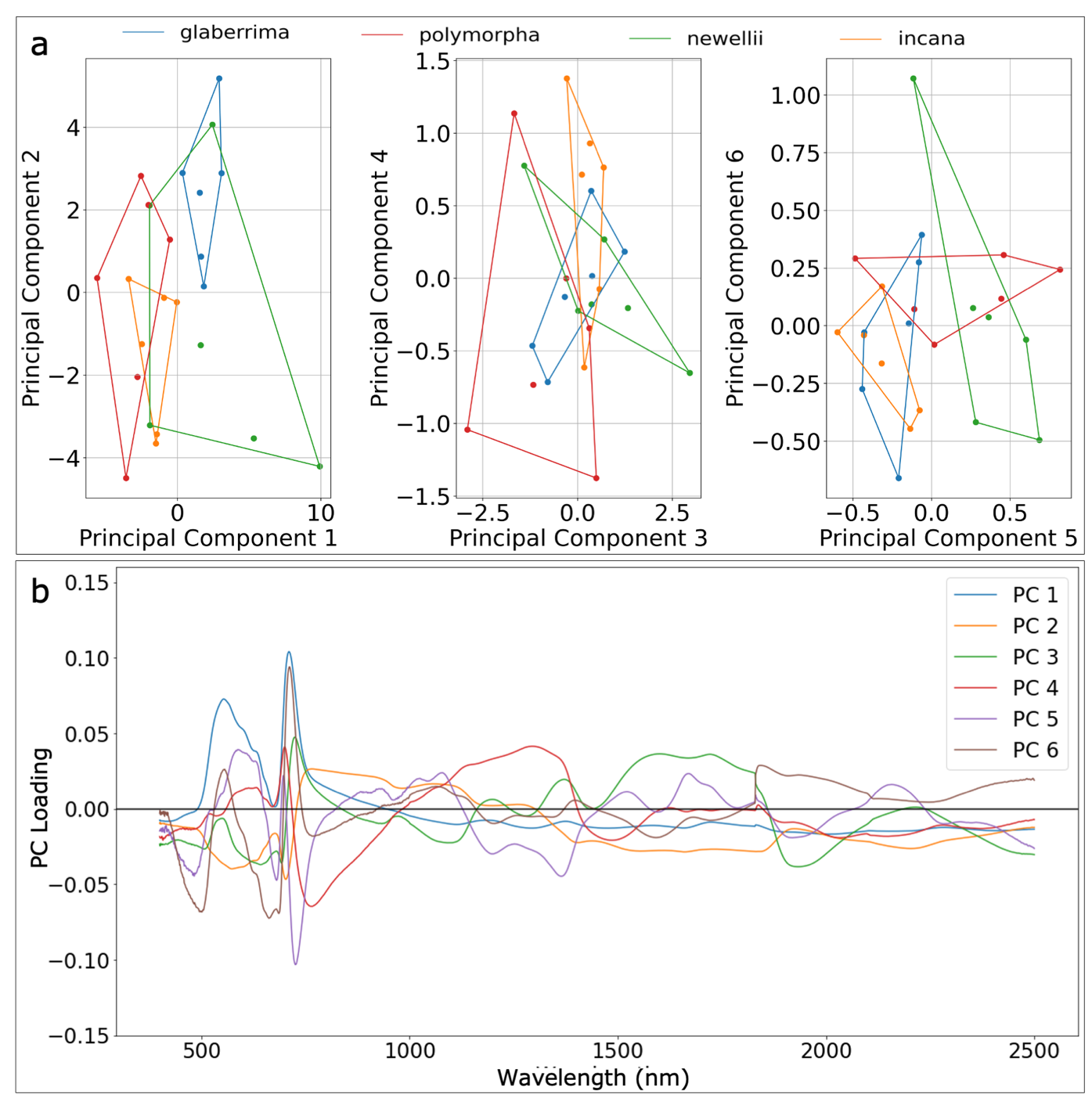
**Figure SI 3:** a) Reflectance data of the four Hawaii Island *Metrosideros polymorpha* varieties plotted in principal component (PC) analysis space. Axes on the three plots represent different PCs. b) PC loadings of the first 6 PC plotted across the VSWIR spectra.

**
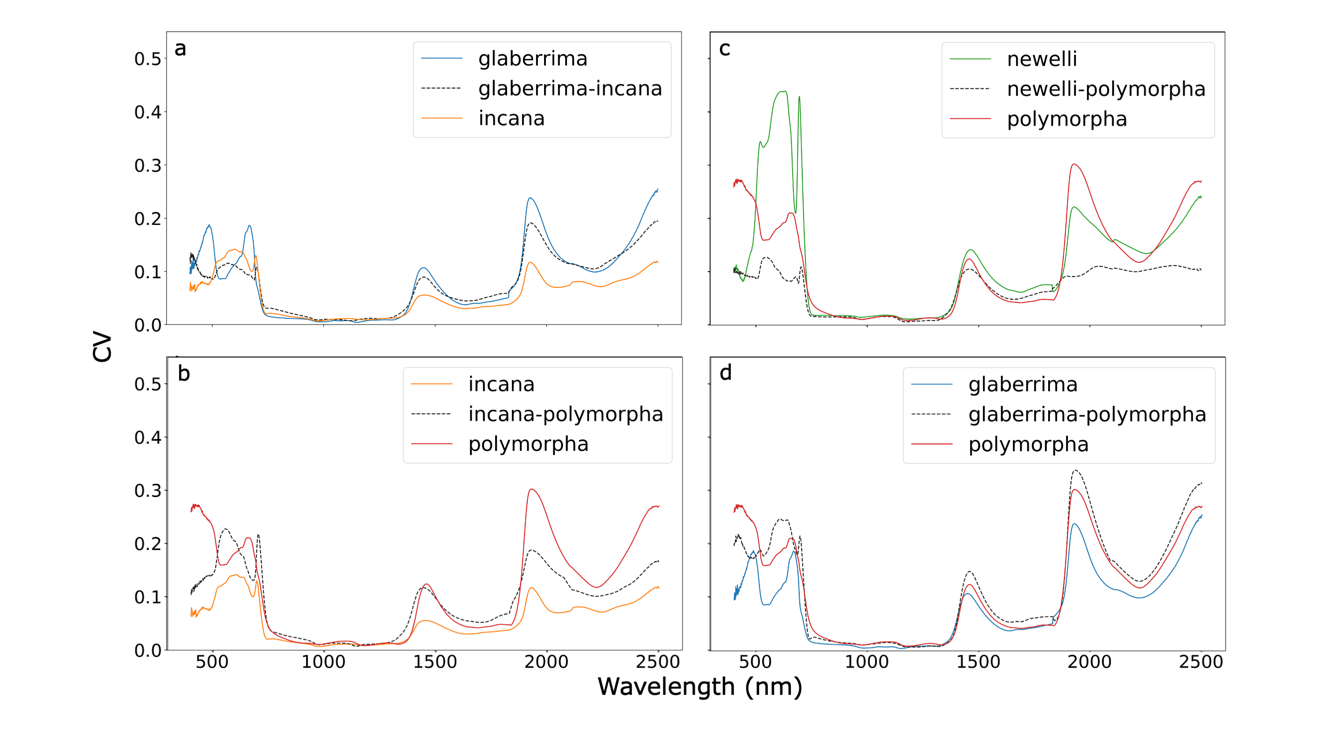
**

**Figure SI 4:** Coefficient of variation (CV) of reflectance of the F1 hybrids alongside their parental taxa: a) glaberrima-incana (GI), glaberrima (G) and incana (I); b) incana-polymorpha (IP), incana (I) and polymorpha (P); c) newellii-polymorpha (NP), newellii (N) and polymorpha (P); d) glaberrima-polymorpha (GP), glaberrima (G) and polymorpha (P).

**Figure SI 5:** Reflectance data of four Hawaii Island *Metrosideros polymorpha* hybrids along with their parent varieties plotted in principal component analysis (PCA) space. PCA was applied separately to each data set grouping.


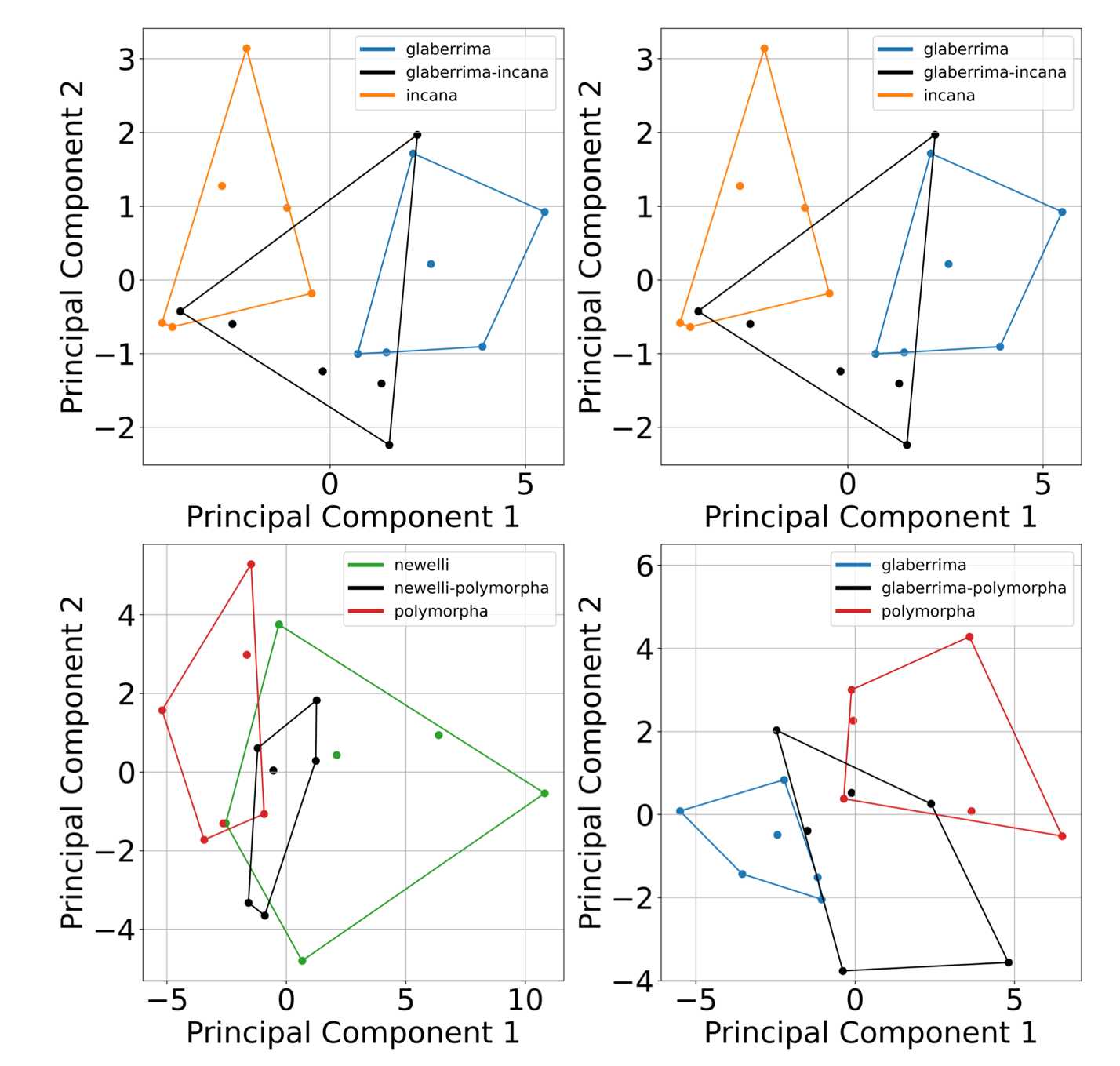

Supplement: Supplementary file 1 — Supplementary file1 (DOCX 4434 KB) [file 442_2023_5374_MOESM1_ESM.docx]
